# Isolation and Identification of Benzochroman and Acylglycerols from Massa Medicata Fermentata and Their Inhibitory Effects on LPS-Stimulated Cytokine Production in Bone Marrow-Derived Dendritic Cells

**DOI:** 10.3390/molecules23092400

**Published:** 2018-09-19

**Authors:** Ya Nan Sun, Seo Young Yang, Young-Sang Koh, Young Ho Kim, Wei Li

**Affiliations:** 1 College of Pharmacy, Chungnam National University, Daejeon 34134, Korea; yanansun@163.com (Y.N.S.); syyang@cnu.ac.kr (S.Y.Y.); 2 School of Medicine and Brain Korea 21 Program, and Institute of Medical Science, Jeju National University, Jeju 690-756, Korea; yskoh7@jejunu.ac.kr; 3 Korean Medicine (KM) Application Center, Korea Institute of Oriental Medicine, Daegu 41062, Korea

**Keywords:** Massa Medicata Fermentata, anti-inflammatory activity, benzochroman, acylglycerol

## Abstract

Massa Medicata Fermentata (MMF), known as Shenqu, is an important traditional Chinese medicine widely used to treat indigestion, vomiting, and diarrhea. In this study, a new benzochroman, 3(*S*)-3,4-dihydro-5,10-di-*β*-d-glucopyranoside-2,2-dimethyl-2*H*-naphtho(2,3-*b*)pyran-3-ol (**1**), and five known galactosyl acylglycerols (**2**–**6**) were isolated from a methanol extract from MMF. In addition, their chemical structures were determined by chemical and spectroscopic methods, which were compared with the previously reported data. Furthermore, the effects of isolated compounds on lipopolysaccharide (LPS)-stimulated bone marrow-derived dendritic cells were investigated. Compounds **1**–**3** exhibited significant inhibitory effects on the LPS-induced production of IL-6 and IL-12 p40, with IC_50_ values ranging from 1.6 to 10.2 μM. Compounds **2** and **3** also exhibited strong inhibitory effects on the LPS-stimulated production of TNF-α with IC_50_ values of 12.0 and 11.2 μM, respectively. The results might provide a scientific basis for the development of the active components in MMF, as well as for novel anti-inflammatory agents.

## 1. Introduction

Massa Medicata Fermentata (MMF), also known as Shenqu, is one of the widely used traditional medicines [1]. MMF is fermented using six traditional medicinal materials: wheat (*Triticum aestivum* L.); red bean (*Vigna umbellata* (Thunb.)); bitter almond (*Prunus armeniaca* L.); sweet wormwood (*Artemisia annua* L.); cocklebur (*Xanthium sibiricum* L.); and water pepper (*Polygonum hydropiper* L.) [1,2,3]. MMF has been widely used for treating indigestion, vomiting, diarrhea, obesity, and related metabolic diseases. Recent pharmacological research showed that MMF demonstrates significant anti-inflammatory activity in an LPS-mediated inflammatory insult, either individually in vitro using RAW264.7 cells or in combination with in vivo using rats [2]. However, the active components have not been reported. The present phytochemical and pharmacological study on MMF searched for anti-inflammatory constituents and unique compounds, which resulted in the isolation of a new benzochroman derivative (**1**) from an MMF methanol extract.

Lipopolysaccharide (LPS) is the most abundant component within the cell wall of Gram-negative bacteria that suppresses inflammatory responses in vivo. It can stimulate the release of several inflammatory cytokines in various cell types, leading to an acute inflammatory response towards pathogens. Interleukin (IL)-6, IL-12, and TNF-*α* is the important pro-inflammatory cytokines that play a significant role in inflammation. Tumor necrosis factor (TNF)-*α* is a pro-inflammatory cytokine produced from monocytes and macrophages after the invasion of various pathogens that attack the host defense mechanisms. TNF-*α* plays a crucial role in host defense and in inflammatory response [4]. Specifically, IL-6 is an important molecule because it exhibits pro- and anti-inflammatory effects, and has been implicated in several inflammatory diseases [5]. IL-12 plays a key role in the initiation and regulation of the cellular immune response [6]. Independent of IL-12 and IL-23, p40 and p80 exhibit specific biological activities [7,8]. On the other hand, dendritic cells (DCs) play a pivotal role in orchestrating immune responses, especially by modulating T-cell function. To identify the MMF anti-inflammatory components, the isolated compounds were investigated for the LPS-induced expression of the pro-inflammatory cytokines IL-6, IL-12 p40, and TNF-α in bone marrow-derived dendritic cells (BMDCs).

## 2. Results and Discussion

### 2.1. Isolation and Structural Elucidation

**Figure 1:**
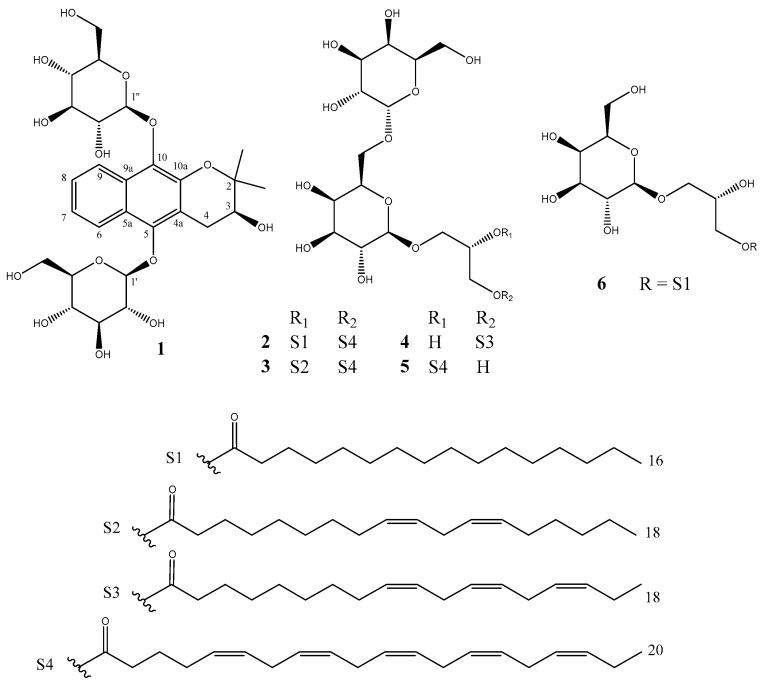
Structures of compounds **1**–**6** from Massa Medicata Fermentata.

Seven compounds (**1**–**6**) were isolated from the methanol extract of the dried MMF, based on spectroscopic data, chemical evidence, and comparison with the previous studies (Figure 1). The HPLC confirmed the purity of the isolated compounds was greater than 95%. Their structures were elucidated as 3(*S*)-3,4-dihydro-5,10-di-*β*-d-glucopyranoside-2,2-dimethyl-2*H*-naphtho(2,3-*b*)pyran-3-ol (**1**), (2*S*)-1-*O*-eicosapentaenoyl-2-*O*-palmitoyl-3-*O*-(*β*-d-galactopyranosyl-6-1*α*-d-galactopyranosyl)-glycerol (**2**) [9], (2*S*)-1-*O*-eicosapentaenoyl-2-*O*-linoleoyl-3-*O*-(*β*-d-galactopyranosyl-6-1*α*-d-galactopyranosyl)-glycerol (**3**) [10], gingerglycolipid A (**4**) [11], (2*S*)-3-*O*-otadeca-9*Z*,12*Z*,15*Z*-trienoylglyceryl-6′-*O*-(*α*-d-galactopyranosyl)-*β*-d-galactopyranoside (**5**) [12], and spongilipid (**6**) [13]. These compounds were isolated from MMF for the first time; in addition, compounds **2**, **3**, and **5** were first isolated from the plant material.

The molecular formula (C_27_H_36_O_14_) of compound **1**, isolated as yellow amorphous powder, was determined on the basis of the positive HR-ESI-MS at *m*/*z* 607.1996 [M + Na]^+^ (calcd. 607.1997). ^1^H and ^13^C-NMR spectra of **1**, which revealed three segments, viz. a benzochroman backbone and two glucosyl groups (Table 1, Supplementary Materials), respectively [14]. Signals corresponding to an ABCD system (1,2-disubstituted aromatic ring) were observed at δ_H_ 8.27 (d, *J* = 7.5 Hz, H-9), 8.25 (d, *J* = 7.5 Hz, H-6), 7.27 (t, *J* = 7.5 Hz, H-7), and 7.19 (t, *J* = 7.5 Hz, H-8). In the ^1^H-NMR spectrum of **1**. Signals of the chroman group were observed at δ_H_ 3.77 (dd, *J* = 8.5, 5.5 Hz, H-3), 3.59 (dd, *J* = 18.2, 8.5 Hz, H-4(a)), and 2.93 (dd, *J* = 18.2, 5.5 Hz, H-4(b)), and two methyl groups at 1.31 (s) and 1.32 (s). Moreover, two *β*-d-glucose units corresponding to the anomeric proton signals at δ_H_ 4.78 (d, *J* = 7.6 Hz, H-1′) and 4.76 (d, *J* = 7.6 Hz, H-1′′) were observed in the ^1^H-NMR spectrum of **1**. The large coupling constants between H-1 and H-2 of the sugar units provided *β*-linkage.

**Figure 2:**
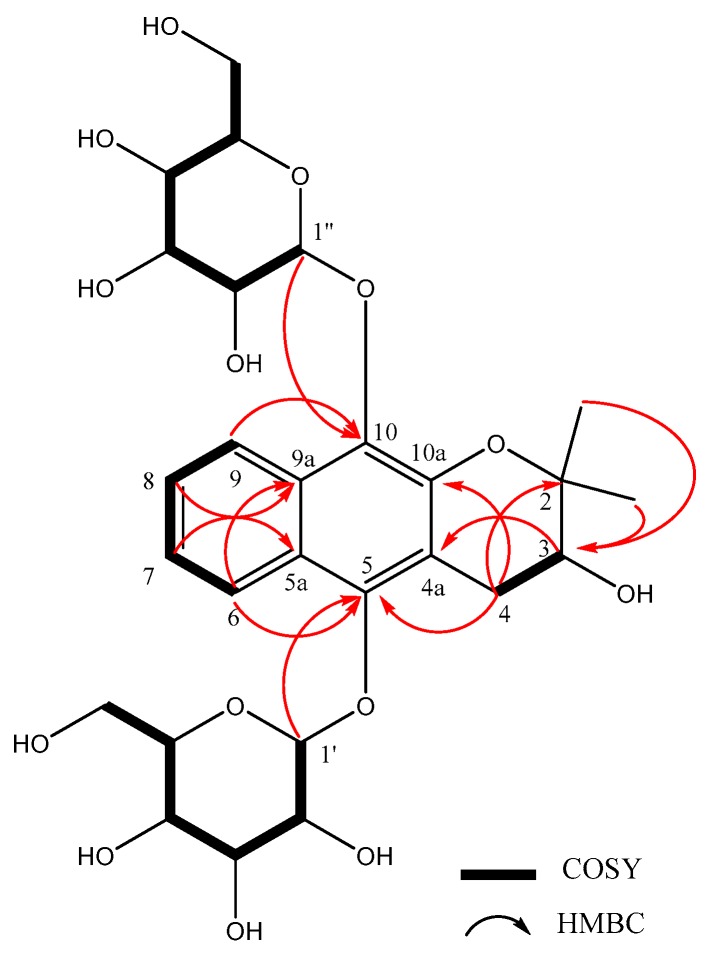
Key ^1^H-^1^H COSY and key heteronuclear multiple bond correlation (HMBC) correlations of compound **1**.

A total 10 aromatic carbons were observed at δ_C_ 148.4 (C-10), 143.4 (C-10a), 137.3 (C-5), 130.0 (C-5a), 126.6 (C-6), 124.7 (C-7), 123.8 (C-9a), 123.6 (C-8), 122.9 (C-9), and 118.4 (C-4a) in the ^13^C-NMR spectrum of **1**. Carbons typical of chroman were observed at δ_C_ 79.0 (C-2), 69.5 (C-3), and 29.1 (C-4), in addition to two methyl groups at δ_C_ 25.2 and 22.7, respectively. From the above data, **1** was possibly a benzochroman glycoside. Correlations between δ_H_ 4.78 (H-1′)/137.3 (C-5) and 4.76 (H-1′′)/148.4 (C-10) were observed in the HMBC spectra of **1**, revealing the attachment of two glucosyl groups to the C-5 and 10 of the backbone (Figure 2, Supplementary Materials). The benzochroman backbone was determined by HMBC correlations at δ_H_ 3.59, 2.53 (H-4)/δ_C_ 143.4 (C-10a) and 137.3 (C-5), δ_H_ 3.77 (H-3)/δ_C_ 118.4 (C-4a), δ_H_ 8.25 (H-6)/δ_C_ 137.3 (C-5) and 123.8 (9a), and δ_H_ 8.27 (H-9)/δ_C_ 148.4 (C-10) and 130.0 (C-5a). The enzymatic hydrolysis of **1** afforded d-glucose as the sugar residues by the comparing with those reported previously by a GC experiment. The enzymatic hydrolysis of **1** afforded aglycone. A positive value for the optical rotation of **1a** (${\left[\alpha \right]}_{D}^{25}$: +15.0 (*c* 0.1, MeOH)) was obtained, revealing the 3*S* configuration of **1** by comparing the physical and spectral data with those reported previously [15]. Based on this result, the structure of **1** was determined to be 3(*S*)-3,4-dihydro-5,10-di-*β*-d-glucopyranoside-2,2-dimethyl-2*H*-naphtho(2,3-b)pyran-3-ol.

### 2.2. Bioassays

To identify the active anti-inflammatory constituents in the MMF methanol extract, the effects of **1**–**6** (at a concentration of 50 μM) on the cell viability of BMDCs was evaluated by a colorimetric MTT assay. Results revealed that the tested compounds did not exhibit cytotoxicity at the concentrations tested (data not shown). Next, the inhibition of the IL-12 p40 production was examined with a concentration of 25 μM. In general, the inhibitory effects were higher than 50%, we considered that the compounds exhibited the inhibitory effects on the production of IL-12 p40. Results revealed that the isolated compounds decreased the production of IL-12 p40 (>50%).

Next, the effects of **1**–**6** on the production of IL-6, IL-12 p40, and TNF-α were examined at various concentrations (1 to 50 μM). 4-(4-Fluorophenyl)-2-(4-methylsulfinylphenyl)-5-(4-pyridyl)-1*H*-imidazole (SB203580), which was an inhibitor of p38 kinase, was used as the positive control [16]. SB203580 inhibited the production of IL-6, IL-12 p40, and TNF-α, with IC50 values of 1.7, 2.5, and 3.6 μM, respectively (Table 2). Compound **1**, which was benzochroman, significantly inhibited the production of IL-6, with IC50 value of 4.6 μM, as well as exhibited strong inhibitory effects on the production of IL-12 p40, with IC50 values of 10.2 μM. However, 1 exhibited the least relevant inhibitory effect on the TNF-α LPS-stimulated production. On the other hand, among galactosyl acylglycerols (**2**–**6**), **2** and **3** significantly inhibited the production of IL-12 p40, IL-6, and TNF-α, with IC_50_ values ranging from 1.6 to 12.0 μM (Table 2). In addition, other compounds exhibited moderate activity at the tested concentrations. The anti-inflammatory activities and structural properties of **2**–**6** also helped to procure information regarding the structure-activity relationship. Interestingly, the effects significantly increased the unsaturated aliphatic substituted acylglycerol, and the activity was proportional to the number of olefinic bonds on each of the aliphatic moieties, suggesting that the number of olefinic bonds played a critical role in the anti-inflammatory activity. These data might be useful in evaluating the SAR of other acylglycerols.

### 2.3. Concluding Remarks

In this study, seven compounds including a new benzochroman (**1**) and five galactosyl acylglycerols (**2**–**6**) were isolated from a methanol extract of MMF. All isolated compounds were evaluated for their inhibitory effects on the production of the LPS-stimulated cytokines (IL-12p40, IL-6, and TNF-α) in BMDCs. To our knowledge, this is the first study on the benzochroman and galactosyl acylglycerol components of MMF and their anti-inflammatory activity. Most of the isolated compounds (**1**–**3**, and **5**) were isolated from the plant material for the first time. The results suggested that benzochromans and galactosyl acylglycerols were bioactive MMF components, which might provide a scientific basis for the development of the active components in MMF, as well as for novel anti-inflammatory agents.

## 3. Materials and Methods

### 3.1. General Information

Optical rotation was determined on a JASCO DIP-370 automatic polarimeter (Jasco, Tokyo, Japan). GC profiles were recorded on a Shimadzu-2010 spectrometer, with an FID detector (Shimadzu, Kyoto, Japan); a detection temperature of 300 °C; an SPB-1 column (0.25 mm i.d. × 30 m); a column temperature of 230 °C; a He carrier gas (2 mL/min); an injection temperature of 250 °C; and an injection volume of 0.5 μL. NMR spectra were recorded on a JEOL ECA 600 spectrometer (^1^H, 600 MHz; ^13^C, 150 MHz; JEOL Ltd., Tokyo, Japan), with tetramethylsilane (TMS) as the internal standard. Heteronuclear multiple quantum correlation (HMQC); heteronuclear multiple bond correlation (HMBC); and ^1^H–^1^H correlation spectroscopy (COSY) spectra were recorded using a pulsed field gradient. ESI-MS spectra were recorded on an Agilent 1200 LC-MSD Trap spectrometer. Melting points were determined on an Electrothermal IA-9200 system (Agilent Technologies, Santa Clara, CA, USA). Column chromatography was performed over silica gel (Kieselgel 60, 70–230, and 230–400 mesh, Merck, Darmstadt, Germany). YMC RP-18 resins, and thin layer chromatography (TLC) was performed using pre-coated silica-gel 60 F_254_ and RP-18 F_254S_ plates (both 0.25 mm, Merck, Darmstadt, Germany), and the spots were detected under UV light and using 10% H_2_SO_4_.

### 3.2. Preparation of Extracts of MMF

Dried MMF was purchased from a herbal company, Naemome Dah (Ulsan, Korea), in April 2016, and identified by Prof. Young Ho Kim, College of Pharmacy, Chungnam National University. A voucher specimen (CNU16105) was deposited at the herbarium of the College of Pharmacy, Chungnam National University (Daejeon, Korea). Dried MMF (5.0 kg) was extracted thrice with MeOH (10 L each for three times) under reflux. After solution concentration, the extract equaled (99.0 g).

### 3.3. Isolation

The extract (99.0 g) was partitioned with EtOAc, affording EtOAc (32.0 g) and H_2_O (67.0 g) fractions. The H_2_O fraction (67.0 g) was subjected to chromatography over a highly porous polymer (Diaion HP-20) and successively eluted with H_2_O and MeOH to give two fractions (Fractions H1 and H2, respectively). Fraction H1 (45.0 g) was subjected to column chromatography over silica gel (8.0 × 30 cm) with a gradient of CHCl_3_–MeOH–H_2_O (30:1:0–1:3:0.1; 1.5 L for each step) to obtain 6 fractions (Fractions H1.1–H1.10). Fraction H1.5 (2.0 g) was separated by RP (1.5 × 60 cm) column chromatography using MeOH–H_2_O (3.5:1–10:1; 1.5 L for each step) as the eluent, affording 4 subfractions (i.e., Fractions H1.5.1–H1.5.4, respectively). Subfraction H1.5.2 (500.0 mg) was further purified on a chromatography column over silica gel (1.0 × 80 cm), affording compound **2** (64.0 mg). Subfraction H1.5.3 (675.0 mg) was purified on a chromatography column over silica gel (1.0 × 80 cm), affording compound **3** (90.0 mg). Fraction H1.8 was subjected to RP (1.5 × 60 cm) column chromatography with a gradient of MeOH–H_2_O (3:1, 10:1, 1.0 L for each step), affording compounds **4** (80.0 mg) and 6 (36.0 mg). Fraction H2 (10.0 g) was separated by column chromatography over silica gel (4.0 × 50 cm) with CHCl_3_–MeOH–H_2_O (20:1:0.1–3:1:0.1; 1.5 L for each step), affording 11 fractions (fractions H2.1–H2.11, respectively). Subfraction H2.8 (500.0 mg) was further purified on a chromatography column over silica gel (CHCl_3_–MeOH–H_2_O: 5.5:1:0.1; 1.5 L; 1.0 × 80 cm) to obtain compound **6** (50.0 mg). Compound **1** (50.0 mg) was isolated from fraction H2.10 by RP (1.0 × 80 cm) column chromatography with MeOH–H_2_O (1:6–1:2; 1.0 L for each step).

*3(S)-3,4-Dihydro-5,10-di-β-d-glucopyranoside-2,2-dimethyl-2H-naphtho(2,3-b)pyran-3-ol* (1): yellow amorphous powder; ${\left[\alpha \right]}_{D}^{25}$: +11.8 (*c* 0.1, MeOH); UV (MeOH): 238 and 280 nm; ^1^H-NMR (methanol-*d*_4_, 600 MHz) and ^13^C-NMR data (methanol-*d*_4_, 150 MHz), see Table 1; HR-ESI-MS: *m*/*z* 607.1996 [M + Na]^+^ (calcd. for 607.1997, C_27_H_36_O_14_).

### 3.4. Enzymatic Hydrolysis

Compound **1** (2.0 mg) was mixed with *β*-glucosidase (2.0 mg) in water (1.0 mL), followed by shaking in a water bath at 37 °C for 12 h. Next, the reaction mixture of 1 was concentrated and subjected to column chromatography over silica gel (1.0 × 15 cm, 40–63 μm), using CHCl_3_–MeOH (15:1, 70 mL), and CHCl_3_–MeOH–H_2_O (7:3:0.5, 60 mL)1a (0.8, affording aglycone mg) and a sugar fraction. Sugar fraction was concentrated to dryness using N_2_. The resulting residues were dissolved in dry pyridine (0.1 mL), followed by the addition of l-cysteine methyl ester hydrochloride in pyridine (0.06 M, 0.1 mL) solution. After heating the reaction mixtures at 60 °C for 2 h, 0.1 mL of the trimethylsilylimidazole solution was added. Heating at 60 °C was continued for an additional 1.5 h. The dried products were partitioned with *n*-hexane and H_2_O (0.1 mL each), and the organic layers were analyzed by gas liquid chromatography on a DB-5 capillary column (0.32 mm × 30 m) with an FID detector; at a column temperature of 210 °C; an injector temperature of 270 °C; detector temperature of 300 °C, and a He carrier gas flow rate of 3 mL/min. Under these conditions, peaks were observed at *t*_R_ (min) = 12.26 and 14.17 for standard sugars l and d-glucose, respectively. Peaks corresponding to the hydrolysate of 1 were detected at *t*_R_ (min) = 14.18, which was identified as d-glucose by comparing with the retention time of the authentic samples after treatment with trimethylsilylimidazole in pyridine.

### 3.5. Cell Culture

BMDCs were grown from wild-type C57BL/6 mice (Orient Bio Inc., Korea) as described in the previous section. (Koo et al., 2012). All animal procedures were approved and performed according to the guidelines of the Institutional Animal Care and Use Committee of Jeju National University (#2010-0028). The mouse tibia and femur were obtained by flushing with Dulbecco’s modified Eagle medium, affording bone marrow cells. The cells were cultured in RPMI 1640 medium containing 10% heat-inactivated fetal bovine serum (FBS; Gibco, New York, NY, USA), 50 μM of β-ME, 2 mM of glutamine supplemented with a 3% J558L hybridoma cell culture supernatant, containing granulocyte/macrophage colony-stimulating factor. The culture medium was replaced with fresh medium every second day. On culture day 6, non-adherent cells and loosely adherent DC aggregates were harvested, washed, and resuspended in 5% FBS-supplemented RPMI 1640.

### 3.6. Cytokine Production Measurements

BMDCs were incubated in 48-well plates in 0.5 mL containing 1 × 10^5^ cells per well, followed by treatment with isolated compounds **1**–**6** at the indicated concentrations (1, 2, 5, 10, 25, and 50 μM) for 1 h before stimulation with 10 ng/mL of LPS from *Salmonella minnesota* (Alexis, New York, NY, USA). Supernatants were harvested 18 h after stimulation. Concentrations of murine TNF-α, IL-6, and IL-12 p40 in the culture supernatants were determined by ELISA (BD PharMingen, San Jose, CA, USA) according to the manufacturer’s instructions. Data are indicated as means ± standard deviation (SD) of at least three independent experiments performed in triplicate.

### 3.7. Statistical Analysis

At least triplicate measurements were independently performed. Data were expressed as the mean ± SD. Statistical significance was determined by one-way analysis of variance followed by Dunnett’s multiple comparison tests, *p* < 0.05.

## Figures and Tables

**Table 1:** ^1^H (600 MHz) and ^13^C-NMR (150 MHz) spectroscopic data of compound **1** (methanol-*d*_4_, δ, ppm, *J*/Hz).

Pos.	δ_H_	δ_C_		δ_H_	δ_C_
**2**		79.0	**1**′	4.78, d, 7.6	106.8
**3**	3.77, dd, 8.5, 5.5	69.5	**2′**	3.54, m	75.9
**4**	(a), 3.59, dd, 18.2, 8.5 (b), 2.93, dd, 18.2, 5.5	29.1	**3′**	3.71, m	78.2
**4a**		118.4	**4′**	3.38, m	71.5
**5**		137.3	**5′**	2.97, m	77.9
**5a**		130.0	**6′**	3.65, m 3.57, m	62.7
**6**	8.25, d, 7.5	126.6	**1′′**	4.76, d, 7.6	106.7
**7**	7.27, t, 7.5	124.7	**2′′**	3.52, m	75.7
**8**	7.19, t, 7.5	123.6	**3′′**	3.71, m	78.0
**9**	8.27, d, 7.5	122.9	**4′′**	3.36, m	71.3
**9a**		123.8	**5′′**	2.99, m	77.8
**10**		148.4	**6′′**	3.64, m 3.54, m	62.5
**10a**		143.4	**CH_3_**	1.32, s	25.2
			**CH_3_**	1.31, s	22.7

Assignments were confirmed by heteronuclear multiple quantum correlation (HMQC) and HMBC experiments, with *J* values (Hz) indicated within parentheses.

**Table 2:** Anti-inflammatory effects of isolated compounds on LPS-stimulated bone marrow-derived dendritic cells.

Compound	IC_50_ (µg/mL) ^a^		
	IL-12 p40	IL-6	TNF-α
**1**	10.2 ± 0.2	4.6 ± 0.1	>100
**2**	7.9 ± 0.2	7.6 ± 0.3	12.0 ± 0.5
**3**	7.6 ± 0.1	1.6 ± 0.1	11.2 ± 0.3
**4**	38.3 ± 0.6	20.6 ± 0.6	49.4 ± 0.7
**5**	29.7 ± 1.1	22.5 ± 0.3	36.0 ± 0.1
**6**	50.4 ± 0.6	>100	>100
SB203580 ^b^	2.5 ± 0.1	1.7 ± 0.2	3.6 ± 0.2

^a^ IC_50_ values for selected compounds are tabulated in the columns of IL-12 p40, IL-6 and TNF-α. Values >100 µM are inactive. ^b^ Positive control.

## Supplementary Materials

Supplementary data associated with this article can be found in the online version. The following are available online. Figure S1: ^1^H-NMR spectrum of compound **1** in CD_3_OD (600 MHz); Figure S2: ^13^C-NMR spectrum of compound **1** in CD_3_OD (150 MHz); Figure S3: HMQC spectrum of compound **1** in CD_3_OD; Figure S4: HMBC spectrum of compound **1** in CD_3_OD; Figure S5: COSY spectrum of compound **1** in CD_3_OD; Figure S6: HR-ESI-MS data of compound **1**; Figure S7: HPLC data of compound **1**.
