# Supplementary material for: Isolation and Identification of Benzochroman and Acylglycerols from Massa Medicata Fermentata and Their Inhibitory Effects on LPS-Stimulated Cytokine Production in Bone Marrow-Derived Dendritic Cells
Source: Molecules. 2018 Sep 19;23(9):2400. doi: 10.3390/molecules23092400 (PMC6225101; doi:10.3390/molecules23092400)
Supplement: Supplementary file 1 [file molecules-23-02400-s001.pdf]

Supplementary Data

Isolation and Identification of Benzochroman and Acylglycerols from Massa Medicata Fermentata and Their Inhibitory Effects on LPS-stimulated Cytokine Production in Bone Marrow-derived Dendritic Cells

Ya Nan Sun[1,†], Seo Young Yang[1,†], Young-Sang Koh[2], Young Ho Kim[1,*], and Wei Li [3,*]

1 College of Pharmacy, Chungnam National University, Daejeon 34134, Republic of Korea; yanansun@163.com (Y.N.S.); syyang@cnu.ac.kr (S.Y.Y.); yhk@cnu.ac.kr (Y.H.K.)

2 School of Medicine and Brain Korea 21 Program, and Institute of Medical Science, Jeju National University, Jeju 690-756, Republic of Korea; yskoh7@jejunu.ac.kr (Y.S.K.)

3 Korean Medicine (KM) Application Center, Korea Institute of Oriental Medicine, Daegu 41062, Republic of Korea; liwei1986@kiom.re.kr (W.L.)

[*]Correspondence: liwei1986@kiom.re.kr; Tel.: +82-53-940-3874 (W.L.); yhk@cnu.ac.kr, Tel.: +82-42-821-5933 (Y.H.K.)

[†] Ya Nan Sun and Seo Young Yang have contributed equally to this work.

Contents

Figure S1. ^{1}H NMR spectrum of compound **1** in CD_3OD (600 MHz)

Figure S2. ^{13}C NMR spectrum of compound **1** in CD_3OD (150 MHz)

Figure S3. HMQC spectrum of compound **1** in CD_3OD

Figure S4. HMBC spectrum of compound **1** in CD_3OD

Figure S5. COSY spectrum of compound **1** in CD_3OD

Figure S6. HR-ESI-MS data of compound **1**

Figure S7. HPLC data of compound **1**

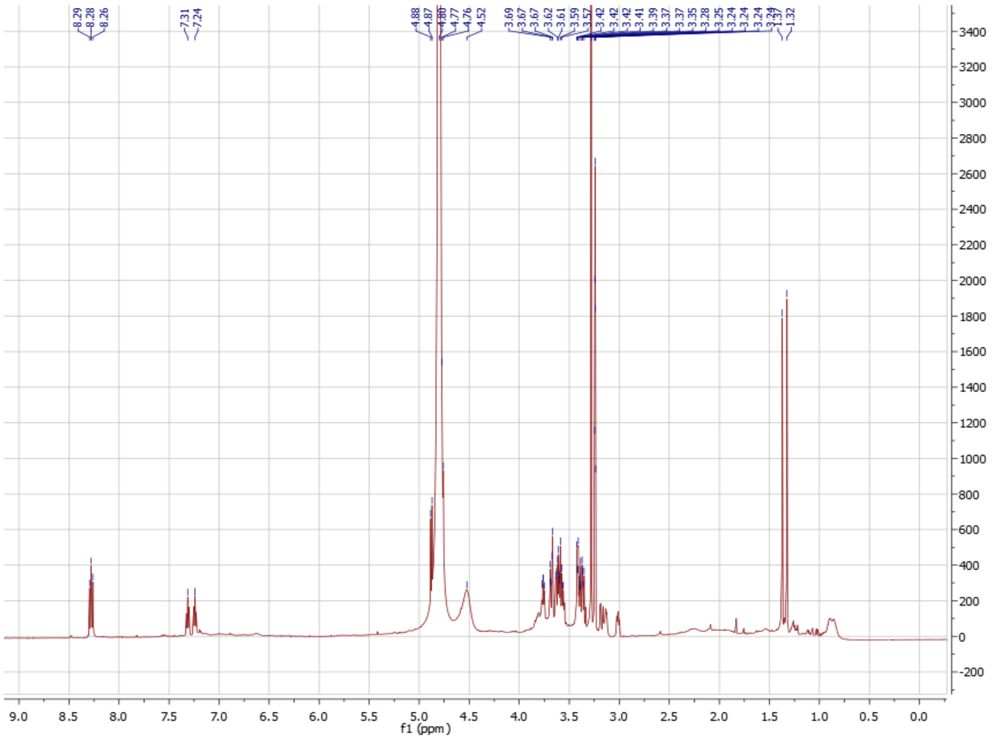

Figure S1. ^{1}H NMR spectrum of compound **1** in CD_3OD (600 MHz)

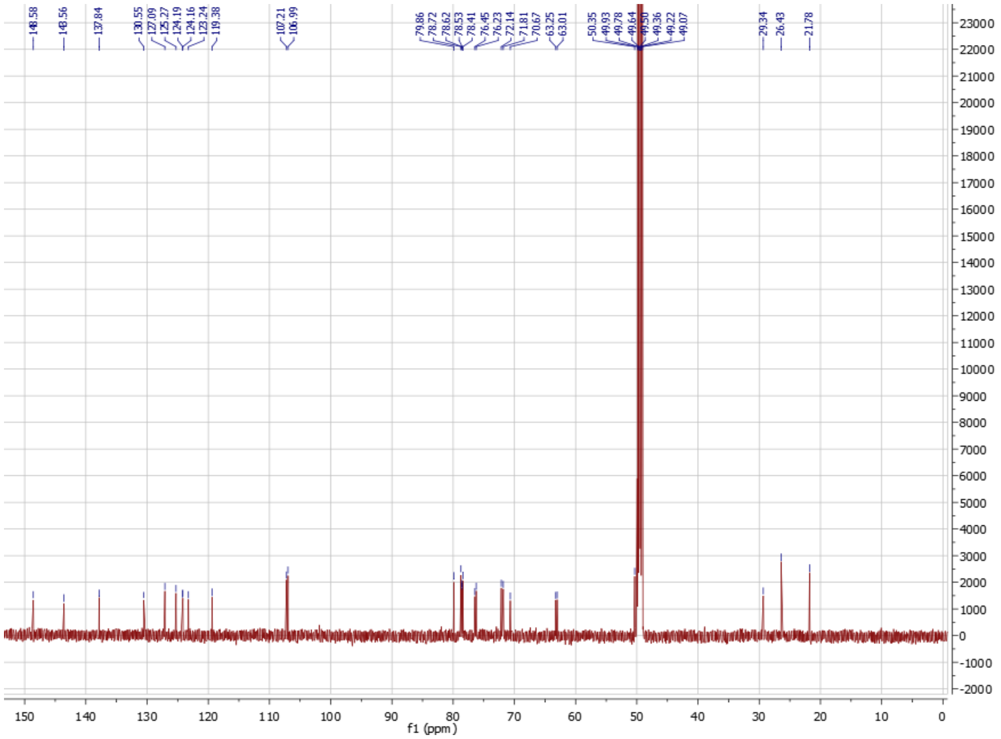

Figure S2. ^{13}C NMR spectrum of compound **1** in CD_3OD (150MHz)

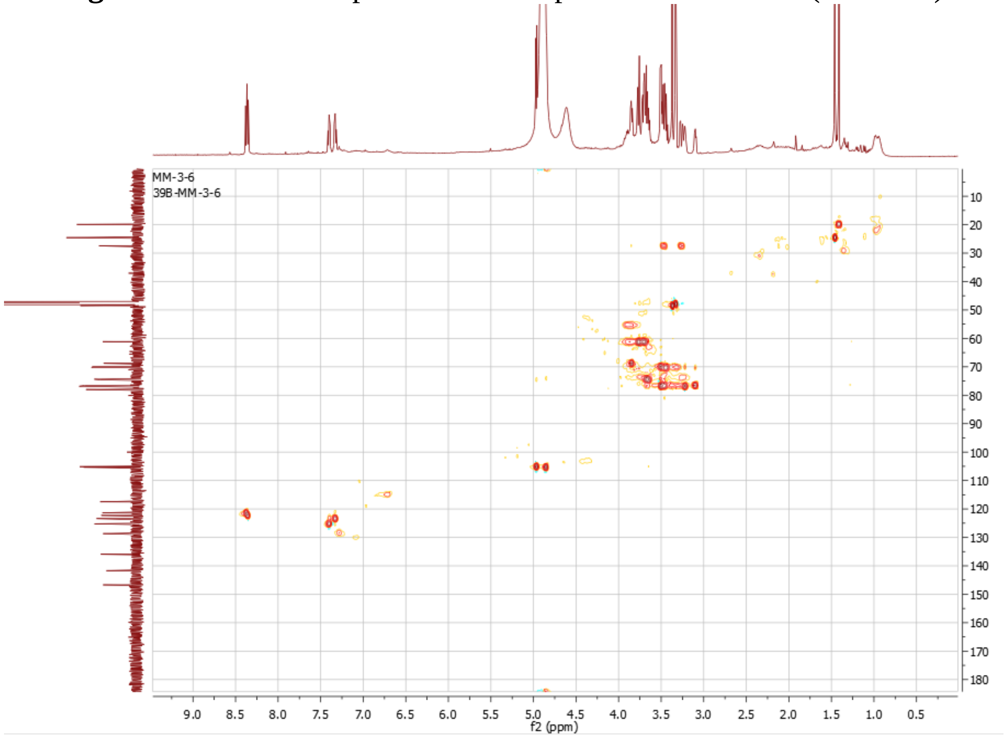

Figure S3. HMQC spectrum of compound **1** in CD_3OD

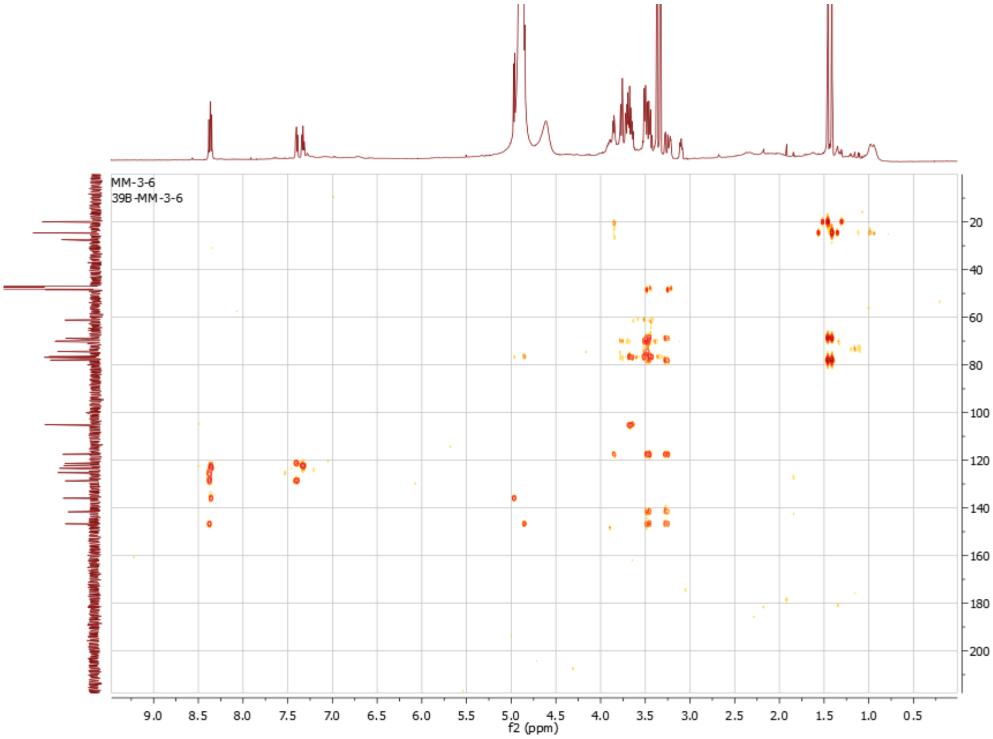

Figure S4. HMBC spectrum of compound **1** in CD_3OD

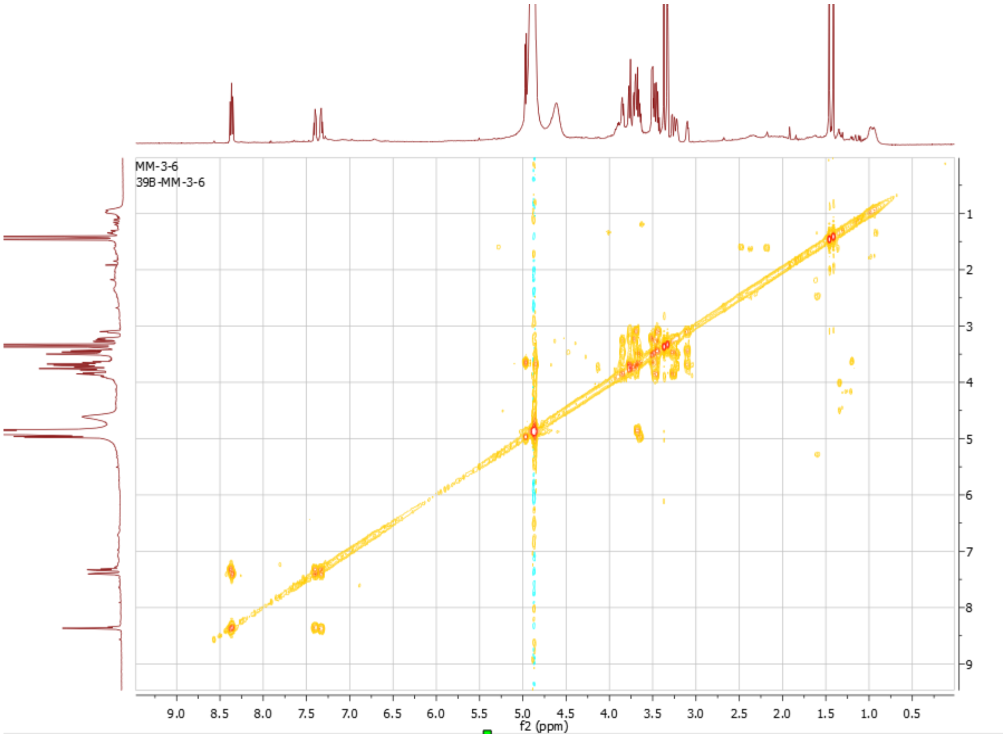

Figure S5. COSY spectrum of compound **1** in CD_3OD

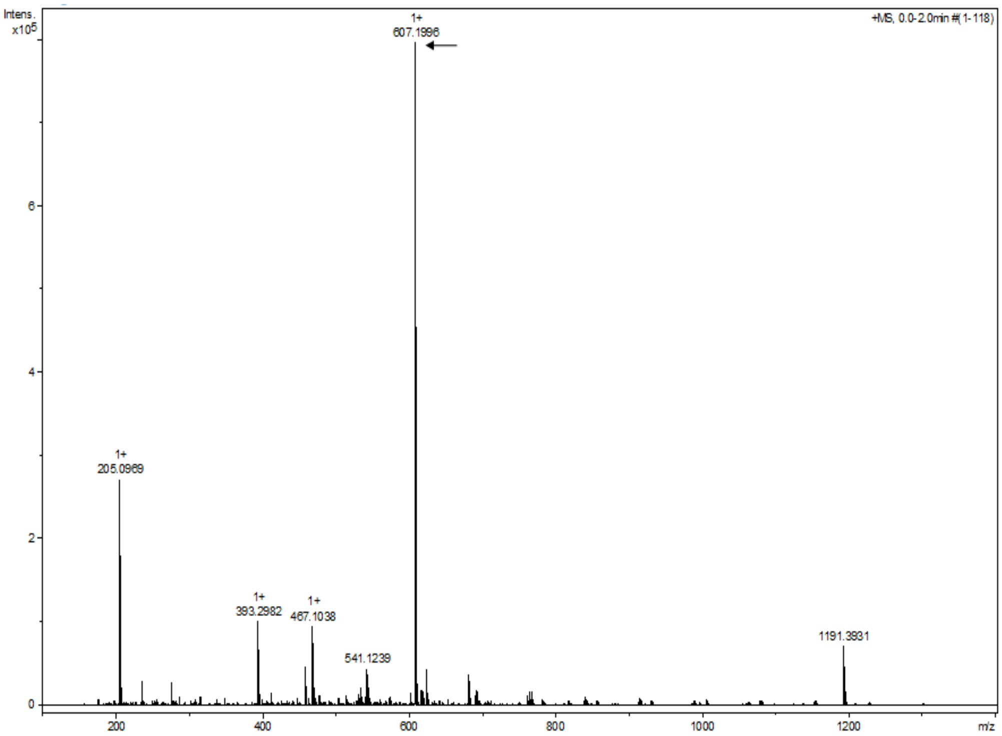

Figure S6. HR-ESI-MS data of compound **1**

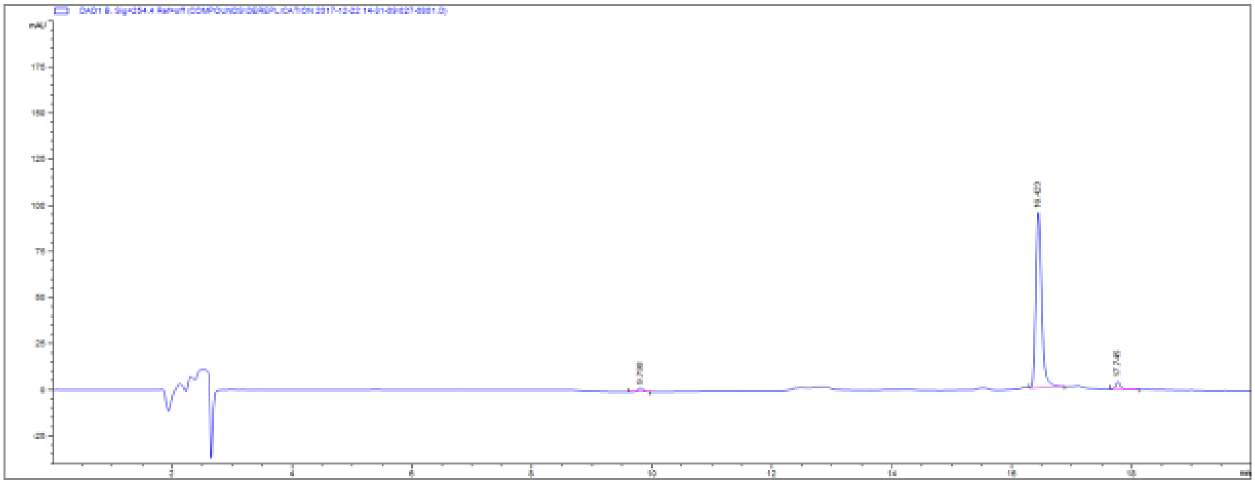

Figure S7. HPLC data of compound **1**

Instrument : Agilent 1260 infinity HPLC system (Agilent Technologies, Santa Clara, CA, USA)

Column : Phenomenex, Luna C18 (2), 5 mm, 100 Å, 150 x 4.6 mm, room temperature

Flow rate: 0.8 mL/min

Detection : UV 254 nm

Time (min)	Water (%)	ACN (%)
0	90	10
5	90	10
15	0	100
20	0	100
